# Dairy product consumption and risk of hip fracture: a systematic review and meta-analysis

**DOI:** 10.1186/s12889-018-5041-5

**Published:** 2018-01-22

**Authors:** Shanshan Bian, Jingmin Hu, Kai Zhang, Yunguo Wang, Miaohui Yu, Jie Ma

**Affiliations:** 1 Department of Nutrition, the Second Hospital of Tianjin Medical University, No. 23 Pingjiang Road, Tianjin, 300211 China; 2 Department of Orthopedics, the Second Hospital of Tianjin Medical University, No. 23 Pingjiang Road, Tianjin, 300211 China; 3 Health Examination Centre, the Second Hospital of Tianjin Medical University, No. 23 Pingjiang Road, Tianjin, 300211 China

**Keywords:** Hip fracture, Diet, Dairy products, Milk consumption, Meta-analysis, Case-control study, Cohort study

## Abstract

**Background:**

Dairy product consumption may affect the risk of hip fracture, but previous studies have reported inconsistent findings. The primary aim of our meta-analysis was to examine and quantify the potential association of dairy product consumption with risk of hip fracture.

**Methods:**

We searched the databases of PubMed and EMBASE for relevant articles from their inception through April 17, 2017. The final analysis included 10 cohort studies and 8 case-control studies. Random-effects models were used to estimate the pooled risk. Subgroup and dose-response analyses were conducted to explore the relationships between the consumption of milk and the risk of hip fracture.

**Results:**

After pooling the data from the included studies, the summary relative risk (RR) for hip fracture for highest versus lowest consumption were 0.91 (95% CI: 0.74–1.12), 0.75 (95% CI: 0.66–0.86), 0.68 (95% CI: 0.61–0. 77), 1.02 (95% CI: 0.93–1.12) for milk, yogurt, cheese, and total dairy products in cohort studies, respectively. Higher milk consumption [Odds ratio (OR), 0.71, 95% CI: 0.55–0. 91] was associated with lower risk of hip fracture for highest versus lowest consumption in case-control studies. After quantifying the specific dose of milk, the summary RR/OR for an increased milk consumption of 200 g/day was 1.00 (95% CI: 0.94–1.07), and 0.89 (95%CI: 0.64–1.24) with significant heterogeneity for cohort and case-control studies, respectively; There was a nonlinear association between milk consumption and hip fracture risk in cohort, and case-control studies.

**Conclusions:**

Our findings indicate that consumption of yogurt and cheese was associated with lower risk of hip fracture in cohort studies. However, the consumption of total dairy products and cream was not significantly associated with the risk of hip fracture. There was insufficient evidence to deduce the association between milk consumption and risk of hip fracture. A lower threshold of 200 g/day milk intake may have beneficial effects, whereas the effects of a higher threshold of milk intake are unclear.

**Electronic supplementary material:**

The online version of this article (10.1186/s12889-018-5041-5) contains supplementary material, which is available to authorized users.

## Background

Hip fracture is the most serious type of osteoporotic fracture. Hip fractures can lead to other comorbidities, increased mortality risk, and enormous social and economic costs [1]. According to recent reports, approximately 1.66 million patients are diagnosed with hip fracture occur each year worldwide [2, 3]. World population surveys have shown that the number of adults older than 60 years old was 841 million in 2013, which is approximately four times as high as that in 1950 (202 million) [4]. The incidence of hip fracture increases linearly with age [5]. The pathogenesis of hip fracture is multifactorial. The main factors contributing to the development of hip fracture are bone mineral density, falls, and lifestyle habits. Lifestyle habits include calcium intake, general nutrition, and exposure to sunlight, physical activity, smoking, and alcohol intake [6–10]. It is known that nutrition, especially dairy product consumption, has an important effect on maintaining bone health.

Dairy products have been hypothesized to help prevent hip fracture because they are a significant source of calcium, proteins, and other bioactive nutrients beneficial for bone health [11]. However, the effects of dairy products on hip fractures have not been established. The benefit of milk, as a main dietary source of calcium in reducing hip fracture risk has yet to be debated. More recently, a study by Sahni et al., [12] indicated that there was a nonsignificant 42% reduction in hip fracture risk in elderly adults who consumed more than 7 milk servings per week, compared with those who consumed less than one serving per week in the Framingham Original Cohort. Michaëlsson et al., [13] found that for every glass of milk consumed per day, women had a significant 9% increase in hip fracture risk, although no association was observed in men. Meanwhile, the data suggested that higher cheese or yogurt intake may reduce hip fracture risk in both men and women [7].

Two previous meta-analyses have been published with results focused only on milk consumption [14, 15]. However, some studies assessed the association between hip fracture and consumption of different types of dairy products, since different types of products contain varying nutrient contents (e.g., milk is rich in lactose, cheese and yogurt can provide lactic acid bacteria). Choosing dairy products like milk, cheese, or yogurt instead of cream can decrease fat, and cholesterol. Therefore, it is important to evaluate the influence of the consumption of different types of dairy products (total dairy products, milk, yogurt, cheese, and cream) on hip fracture risk.

## Methods

### Search strategy

**Fig. 1:**
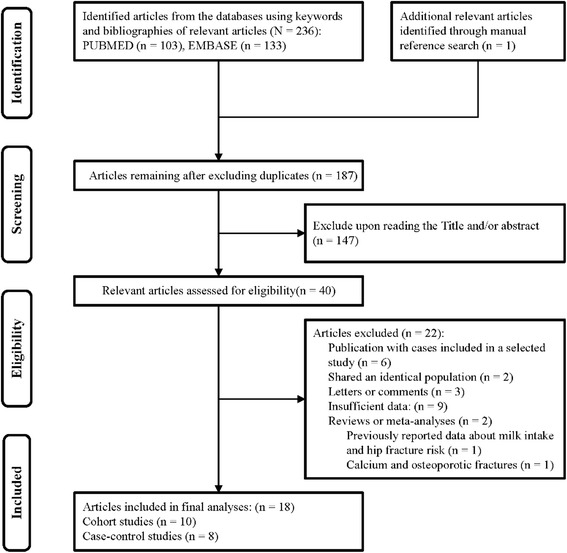
Search strategy and selection of studies for this meta-analysis

The primary aim of our meta-analysis was to examine and quantify the potential association of dairy product consumption with risk of hip fracture. Searches were performed on PubMed and EMBASE databases from their inception to April 17, 2017. The search terms used were as follows: “hip fracture” (or “subtrochanteric fracture” or “trochanteric fracture” or “intertrochanteric fracture” or “femoral neck fracture”) and “dairy products” (or “milk” or “cheese” or “yogurt” or “cream”). No language restrictions were applied in the search strategy. An additional article [16] was identified through the bibliographies of relevant reviews. Figure 1 and Additional file 1 provides detailed search terms and search strategies for both databases.

### Eligibility criteria

To identify eligible studies, two independent investigators (S.B.and J.H.) performed an initial screening of all titles and abstracts, and then assessed the full text of all relevant studies in detail. Articles were included in this meta-analysis if they met the following criteria: (1) cohort or case-control study design; (2) studies that evaluated and clearly defined exposure to total dairy products, milk, cheese, yogurt, or cream; (3) studies with the risk of hip fracture as the outcome of interest; and (4) studies reporting odds ratio (OR) or relative risk (RR) with 95% confidence intervals (CI) for the association between dairy product exposure and risk of hip fracture. Studies had to define hip fracture using the criteria based on the International Classification of Diseases, 10th revision (ICD-10) or medical records. If there were multiple publications from the same study, we selected the most recent study for the meta-analysis. Studies were excluded if they provided insufficient data, such as letters, reviews, comments, or animal studies. Two previous meta-analyses had been published with results focused only on milk consumption. The meta-analysis conducted by Bischoff-Ferrari et al., [14] was excluded, because the report contained duplicated data. The meta-analysis conducted by Kanis et al., [15] was included in our meta-analysis. The flow diagrams of the selection process and results are shown in Fig. 1.

### Data processing and quality assessment

Two authors (Y.W. and K.Z.) independently extracted the following information from each included study: author name, research region, publication year, study design, study name, subjects (number of cases), sex, mean/median age of the study individuals, duration of follow-up for cohort studies, exposure and quantity of intake, dietary assessment method, the maximally adjusted risk estimates with 95% CI for the highest versus the lowest category of consumption, and adjustment for confounders in analyses.

In the dose-response meta-analysis of the relationship between dairy products and hip fracture risk, the number of cases and participants or person-years, the mean or median dairy product consumption for each exposure category, and the RR/OR and its variance estimate for three or more quantitative exposure categories were compiled from the included studies. The median level of milk consumption (g/day) for each exposure category was presented with the relevant RR/OR and corresponding 95% CI. We used standard conversions from the Food Standards Agency to convert glasses/d to g/d (1 glass = 200 mL) for relevant studies [17, 18]. Assumptions were used to convert ml/d to approximate g/d [19]. If dairy products were reported as servings, drinks, or times per day/week/month instead of quantity, the following average amounts were used to represent a serving: 177 g for total dairy products, 244 g for milk and yogurt, and 43 g for cheese consumption [20, 21].

Two investigators (M.Y. and K.Z.) independently assessed the quality of the 17 included studies (Excluding the meta-analysis [15] as previously mentioned) using the Newcastle-Ottawa scale (NOS) [22]. This scale scores studies on three categories (selection, comparability of study groups, and the outcome of interest). A study can be awarded a maximal score of 9, which represents the highest quality study.

### Statistical analysis

Effect sizes were estimated with RR in cohort studies and OR in case-control studies. Cohort and case-control studies were pooled separately in our meta-analysis. We quantified the association of dairy product consumption with hip fracture risk using random-effects models [23].

Subgroup and meta-regression analyses were performed to assess potential sources of heterogeneity stratified by some of the baseline characteristics, such as study quality, region, sex, number of cases, duration of follow-up for cohort studies, age, and adjustment for potential confounders. We also investigated the influence of different types of dairy product consumption, including total dairy products, yogurt, cheese, and cream.

In the dose-response meta-analysis, we used the method proposed by Greenland and Longnecker [24] to estimate the dose-response trend of the relationship between dairy product consumption and hip fracture risk. We applied random effects models [25] to estimate the summary RR or OR within each study.

Between-study heterogeneity was evaluated using both the Q and I^2^ statistics. A Q statistic with *P* < 0.10 indicated heterogeneity, whereas I^2^ values of 0%, 25%, 50%, and 75% represented no, low, moderate, and high heterogeneity, respectively [25]. Publication bias was considered by visual inspection of the contour-enhanced funnel plot symmetry as well as by Egger’s test [26] and Begg’s test [27]. Furthermore, Duval’s non-parametric trim-and-fill procedure was performed to adjust for the number of missing studies and estimate possible publication bias [28]. Meta-analyses were conducted with R version 3.1.2 (The R Foundation for Statistical Computing, Vienna, Austria), using the following packages: meta, foreign, dosresmeta, Hmisc, survival, SparseM, and rms. All statistical tests were two-sided, and *P* values <0.05 indicated statistical significance.

## Results

### Literature search

Figure 1 shows the search strategy and selection of studies for our meta-analysis on dairy product consumption and the risk of hip fracture. We identified one additional article by manually searching the reference lists from the included studies [16]. A total of 18 articles [12, 13, 15, 16, 29–42] were included in the present meta-analysis (Additional file 2).

### Characteristics of included studies

Table 1 shows the detailed baseline characteristics of the included studies. A total of 18 observational studies (10 cohort studies [12, 13, 15, 16, 33–37, 42] and 8 case-control studies [29–32, 38–41]) involving 381,987 participants were included in the final analysis. The 10 cohort studies were published between 1997 and 2014, with a total of 8613 hip fracture events, and 363,557 participants. The length of follow-up ranged from 3 to 22 years. Eight cohort studies included both sexes. One cohort study included only male individuals and the remaining cohort study recruited only females. Four cohort studies were conducted in the USA, 4 were conducted in Europe, 1 was conducted in Asia, and 1 was a meta-analysis of a multicenter study including participants from Europe, Australia, and Canada. The 8 case-control studies were published between 1992 and 2010, and included 3815 hip fracture cases and 6415 controls. Geographic regions of the case-control studies included Europe (*n* = 3), Australia (*n* = 1), USA (n = 1), and Asia (n = 3).

**Table 1:** Baseline characteristics of studies included in the meta-analysis

First author	Publication year	Region	Study design	Study name	Subjects (cases)	Sex	Mean/median age (years)	Follow up period (years)	Diet assessment	Exposure	Quantity	OR/RR (95% CI)	Ascertainment method of hip fracture	Adjustment for confounders
Feskanich	2014	USA	cohort study	The Nurses’ Health Study (NHS); the Health Professionals Follow-up Study (HPFS)	96,927 (1716)	M/F	F (30–55) M (40–75)	22	FFQ	Milk	≥ 4 vs. 1 glass/day	Males 1.21(0.86–1.64) Females 1.01(0.78–1.31)	Self -reporting	Age, questionnaire cycle, adult milk consumption, calcium supplements, vitamin D supplements, retinol from supplements, total protein, alcohol and caffeine intakes, total energy intake, physical activity, BMI, smoking, use of thiazide diuretics, use of furosemide diuretics and oral steroids (men only], use of hormone replacement therapy, incident diagnoses of osteoporosis and cancer, teenage measures
Michaëlsson	2014	Sweden	cohort study	The Swedish Mammography Cohort; the Cohort of Swedish Men	106,772 (5425)	M/F	56.5 (39–79)	F: 20.1 M:11.2	FFQ	Milk	≥ 600 vs. < 200 g/day	Males: 1.01 (0.85–1.20) Females: 1.60(1.39–1.84)	Registers	Age, BMI, height, total energy intake, total alcohol intake, healthy dietary pattern, calcium and vitamin D supplementation, ever use of cortisone, educational level, living alone, physical activity level estimated as metabolic equivalents, smoking status, and Charlson comorbidity index; for women only, use of estrogen replacement therapy and nulliparity
										Yogurt	≥ 400 vs. < 1 g/day	Males: 0.75 (0.63–0.90) Females: 0.70 (0.57–0.86)		
										Cheese	≥ 60 vs. < 20 g/day	Males: 0.75 (0.62–0.92) Females: 0.64 (0.55–0.74)		
Sahni	2014	USA	cohort study	The Framingham Original Cohort	764 (97)	M/F	76.9 (68–96)	11.6	Validated FFQ	Milk	≥ 7 vs. ≤ 1 servings/week	0.58 (0.31–1.06)	Self-reporting confirmed by review of medical records and radiographic and operative reports	Age, sex, weight, height, total energy intake, current cigarette smoking, calcium supplement use, vitamin D supplement use
										Yogurt	> 0 vs. 0 servings/week	1.09 (0.65–1.81)		
										Cheese	> 1 vs. ≤ 1 servings/week	0.72 (0.48–1.08)		
										Cream	≥ 3 vs. < 1 servings/week	1.04 (0.59–1.86)		
Benetou	2011	European	cohort study	The Cancer and nutrition (EPIC) study	29,122 (275)	M/F	64.3 (60–86)	8	Validated FFQ	Total dairy products	Yes vs. no	1.02 (0.93–1.12)	Both registers and self-reporting	Sex, age, BMI, height, educational level, smoking status, physical activity at leisure, supplement use, history of diabetes at enrolment, total energy intake
Feart	2013	France	cohort study	The Three-City (3C) study	1482 (57)	M/F	75.9 (67.7–94.9)	8	FFQ and a 24-h dietary recall	Dairy products	Highest vs. lowest category	1.05 (0.60–1.85)	Self-reporting	Each individual food group component of the Mediterranean diet score, age, gender, physical activity, total energy intake, educational level, marital status, BMI, osteoporosis treatment, calcium and/or vitamin D treatment
										Milk	Highest vs. lowest category	0.86 (0.50–1.49)		
										Yogurt	Highest vs. lowest category	0.90 (0.50–1.61)		
										Cheese	Highest vs. lowest category	0.78 (0.44–1.39)		
Cumming	1997	USA	cohort study	Study of Osteoporotic Fractures (SOF Study)	9704 (306)	F	71 (65+)	6.6	Validated FFQ	Milk	≥ 3 vs. rarely/never glasses/day	0.90 (0.50–1.70)	Self-reporting	Age, clinic, weight, history of osteoporosis, history of fractures since age 50, fall in past 12 months, protein intake, caffeine intake, recreational physical activity, take walks for exercise, impaired low frequency contrast sensitivity, estrogen replacement therapy, thiazide use, use of calcium and Vitamin D supplements, use of Turns antacid tablets
Fujiwara	1997	Japan	cohort study	the Adult Health Study (AHS)	4573 (55)	M/F	58.5	14	Standardized questionnaire	Milk	≥ 5 vs. ≤ 1 times/week	0.54 (0.25–1.07)	Registers	Age, alcohol, BMI, prevalent vertebral fracture, number of children, age at menarche
Meyer	1997	Norway	cohort study	the National Health Screening study	39,787 (213)	M/F	47.1 (42.9–65.9)	11.4	FFQ	Milk	≥ 5 vs. < 1 glasses/day	Males: 0.46 (0.22–0.98) Females: 0.83 (0.44–1.56)	Self-reporting confirmed by review of medical records and radiographic and operative reports	Age, body height, BMI, self-reported physical activity at work and during leisure time, diabetes mellitus, disability pension, marital status, smoking
Owusu	1997	USA	cohort study	The Health Professionals Follow-up Study	43,063 (56)	M	54 (40–75)	8	Validated FFQ	Milk	2.5 vs. ≤1 glass/week	0.97 (0.39–2.42)	Self-reporting	Age, alcohol consumption, smoking, BMI, physical activity, total energy intake
Kanis	2004	Europe, Australia, and Canada	Meta-analysis of cohort study	The European Vertebral Osteoporosis Study (EVOS);The Canadian Multicentre Osteoporosis Study (CaMos);The Dubbo Osteoporosis Epidemiology Study (DOES);The Rotterdam Study;The Sheffield Study;The Gothenburg study	39,563 (413)	M/F	66.7(58.9–80.0)	3–8	NR	Milk	'Highest vs. lowest category of consumption	Males:0.66 (0.39–1.12) Females: 0.92 (0.69–1.22)	Both registers and self-reporting	NR
Jha	2010	India	case-control study	NR	200 (100)	M/F	65.2	NR	Standardized questionnaire	Milk,	> 1 vs. ≤ 1 glass/day	0.30 (0.13–0.72)	Self-reporting	NR
										Cheese	> 1 vs. ≤ 1 servings/week	0.48 (0.24–0.93)		
										Yogurt	> 2 vs. ≤ 2 cups/week	0.77 (0.39–1.51)		
Lan	2010	Taiwan	case-control study	NR	725 (228)	M/F	(60+)	NR	Standardized questionnaire	Milk	≥ 6 vs. none or <1 drink/week	0.58 (0.37–0.91)	Self-reporting	Socio-demographic, disease history, self-assessed health, anthropometry and health habits, diet habits, injury-related experience, physical functioning, cognitive and other functioning, physical performance, female reproductive history, bone mineral density
Jitapunkul	2001	Thai Chinese	case-control study	NR	120 (60)	F	71.4	NR	Structured questionnaire	Milk	Yes vs. no	0.26 (0.09–0.76)	Self-reporting	No regular intake of milk, low number of pregnancies, thin body appearance, low serum calcium
Kanis	1999	Southern Europe	case-control study	The MEDOS study	1862 (730)	M	74	NR	Standardized questionnaire	Milk	Highest vs. lowest category of consumption	0.82 (0.61–1.11)	Registers	BMI, recreational physical activity, consumption of tea, alcohol consumption, coffee consumption, smoking, sunlight exposure
										Cheese	Yes vs. no	0.75 (0.49–1.14)		
Johnell	1995	Southern Europe	case-control study	The MEDOS Study	5618 (2086)	F	77.8	NR	Standardized questionnaire	Milk	Highest vs. lowest category	0.71 (0.58–0.87)	Registers	Mental score, BMI, menarche, menopause, sunlight exposure, tea consumption
Tavani	1995	Italy	case–control study	NR	960 (241)	F	63.3 (45–74)	NR	Standardized questionnaire	Milk	> 7 vs. < 7 drinks/week	1.00 (0.60–1.60)	Registers	Age, education, BMI, estrogen replacement therapy
										Cheese	> 6 vs. < 4 portions/week	1.00 (0.70–1.50)		
Cumming	1994	Australia	case-control study	NR	416 (209)	M/F	(65–100)	NR	Standardized questionnaire	Dairy product	Highest vs. lowest category	1.70 (0.50–5.40)	Registers	Age, sex, country of birth, mental state score, psychotropic drug use, smoker status, work in the house and garden, weight
Nieves	1992	USA	case-control study	NR	329 (161)	F	(50–103)	NR	Validated FFQ	Milk	≥ 7 vs. no glasses/week	1.10 (0.63–1.94)	Registers	Hospital site, age and BMI, smoking status, alcohol consumption

Abbreviations: *BMI*, body mass index (calculated as weight in kilograms divided by height in meters squared); *F*, Female; *M*, male; *FFQ*, food frequency questionnaire; *NR*, not reported

### Milk consumption and risk of hip fracture

Nine cohort studies [12, 13, 15, 16, 33, 34, 36, 37, 42] and 7 case-control studies [29–32, 38, 39, 41] reported the association between milk consumption and risk of hip fracture.

#### Meta-analysis of cohort studies

**Fig. 2:**
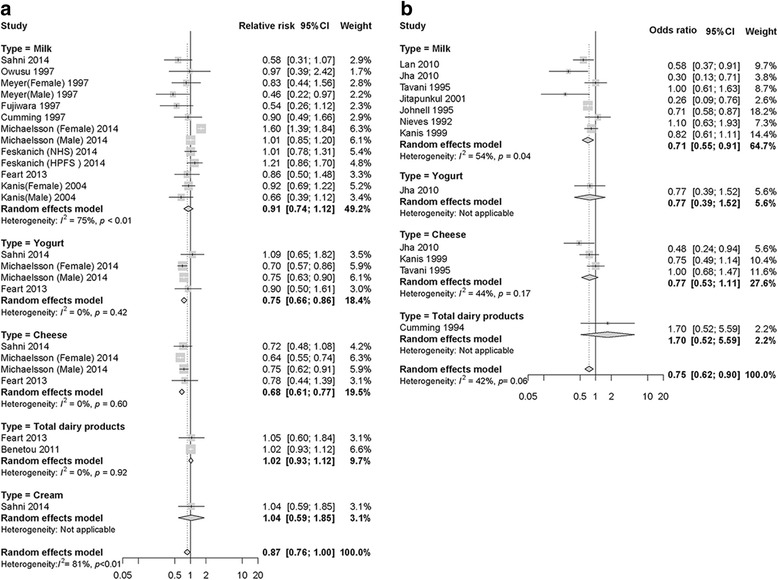
Relative risks of hip fracture for the highest compared with the lowest categories of dairy product consumption. **a** Collection of pooled data from cohort studies; **b** Collection of pooled data from case-control studies. The gray box indicates the 95% confidence intervals (CIs). The size of the square around each effect estimate indicates the weight of the individual study

For cohort studies, the pooled RR for highest versus lowest category of milk consumption and hip fracture risk was 0.91 (95% CI: 0.74–1.12, I^2^ = 75.0%, P_heterogeneity_ < 0.01) (Fig. 2a), indicating that milk consumption had no association with hip fracture risk. The analyses producing the pooled estimates indicated heterogeneity.

**Fig. 3:**
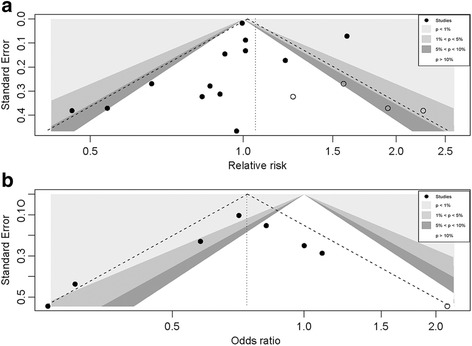
Contour-enhanced funnel plot of Milk consumption and hip fracture risk. **a** Data are collected from cohort studies; **b** Collection of data from case-control studies. Each dot indicates a different study

The contour-enhanced funnel plot demonstrated asymmetry (Fig. 3a). However, Egger’s test (*P* = 0.81) and Begg’s test (*P* = 0.30) indicated no publication bias with regard to milk intake and hip fracture risk. We used the trim-and-fill method to confirm robustness of the results. There were no significant changes to the results after using the trim-and-fill method when including four missing articles (adjusted random effects summary RR: 1.06, 95% CI: 0.91–1.23).

#### Meta-analysis of case-control studies

The case-control studies indicated that participants in the highest categories of milk consumption had a 29% reduction in the risk of hip fracture (OR = 0.71, 95%CI: 0.55–0.91, I^2^ = 54%, P_heterogeneity_ = 0.04) (Fig. 2b). Pooled estimate analyses indicated heterogeneity.

For case-control studies, the contour-enhanced funnel plot showed asymmetry (Fig. 3b). There were no significant changes to the results after using the trim-and-fill method when including one missing article (adjusted random effects summary OR: 0.74, 95% CI: 0.57–0.97). The trim-and-fill estimates should be interpreted with great caution due to the limitations inherent to the methods used.

#### Quality study, subgroup, and meta-regression analyses

The quality of the 17 included studies (Excluding the meta-analysis study [15] as discussed previously) using the Newcastle-Ottawa scale (NOS) [22] is shown in Tables 2, and 3.

**Table 2:** Quality assessment of the included cohort studies

Newcastle-Ottawa Scale for assessing the quality of cohort studies in meta-analysis											
			Selection				Comparability	Outcome			
Study			Representativeness of the exposed cohort	Selection of the non-exposed cohort	Ascertainment of exposure	Demonstration that the current outcome of interest was not present at start of study	Comparability of cohorts on the basis of the design or analysis	Assessment of outcome	Was follow-up long enough for outcomes to occur	Adequacy of follow up of cohorts	Quality score
1	Feskanich	2014		★	★	★	★★		★	★	7
2	Michaëlsson	2014	★	★	★	★	★★	★	★	★	9
3	Sahni	2014	★	★	★	★	★★	★	★	★	9
4	Feart	2013	★	★		★	★★		★	★	7
5	Benetou	2011	★	★		★	★★		★	★	7
6	Cumming	1997	★	★		★	★★		★	★	7
7	Fujiwara	1997	★	★	★	★	★★	★	★	★	9
8	Meyer	1997	★	★	★	★	★★	★	★	★	9
9	Owusu	1997	★	★		★	★★		★	★	7

A study can be awarded a maximum of one star for each numbered item within the Selection and Outcome categories. A maximum of two stars can be given for Comparability

**Table 3:** Quality assessment of the included case-control studies

Newcastle-Ottawa Scale for assessing the quality of case control studies in meta-analysis											
			Selection				Comparability	Outcome			
Study			Is the case definition adequate	Representativeness of the cases	Selection of controls	Definition of controls	Comparability of cases and controls on the basis of the design or analysis	Ascertainment of exposure	Same method of ascertainment for cases and controls	Non-Response rate	Quality score
1	Jha	2010				★	★★		★	★	5
2	Lan	2010				★	★★		★		4
3	Jitapunkul	2001		★		★	★★		★		5
4	Kanis	1999	★	★		★	★★	★	★	★	8
5	Johnell	1995	★	★		★	★★	★	★	★	8
6	Tavani	1995	★	★		★	★★	★	★	★	8
7	Cumming	1994	★	★	★	★	★★	★	★		8
8	Nieves	1992	★	★		★	★★	★	★	★	8

A study can be awarded a maximum of one star for each numbered item within the Selection and Outcome categories. A maximum of two stars can be given for Comparability

In subgroup and meta-regression analyses, the null association between milk consumption and hip fracture risk was consistently observed in the subgroup analysis of 9 cohort studies [12, 13, 15, 16, 33, 34, 36, 37, 42] stratified by different factors (Table 4), except for studies that did not adjust for total energy intake. In addition, there was an inverse association between calcium and vitamin D supplements and hip fracture risk in cohort studies. An inverse association between milk consumption and hip fracture risk was consistently observed in case-control studies (Fig. 2b); subgroup analysis showed that milk consumption had no association with hip fracture risk in studies from the USA.

**Table 4:** Subgroup analyses comparing milk intake and hip fracture risk for case-control and cohort studies

	Cohort studies (*n* = 9)						Case-control studies (*n* = 7)					
	*n*	RR	95% CI	*I*^2^ (%)	*P* ^a^	*P* ^b^	*n*	OR	95% CI	*I*^2^ (%)	*P* ^a^	*P* ^b^
Study quality												
Score ≥ 8	4	0.98	0.75–1.27	90.0	<0.01	0.90	4	0.80	0.67–0.95	10.0	0.34	0.05
Score < 8	4	1.03	0.86–1.24	0.0	0.82		3	0.42	0.25–0.71	35.0	0.21	
Region												
USA	4	1.00	0.96–1.03	0.0	0.84	0.44	1	1.10	0.63–1.94	NA	NA	0.04
Europe	3	0.98	0.68–1.41	86.0	<0.01		3	0.77	0.65–0.90	0.0	0.39	
Asia	1	0.54	0.26–1.12				3	0.42	0.25–0.71	35.0	0.21	
Sex												
Male	5	0.91	0.70–1.19	48.0	0.10	0.68	1	0.82	0.61–1.11	NA	NA	0.28
Female	6	1.07	0.78–1.47	81.0	<0.01		4	0.78	0.53–1.14	58.0	0.07	
Both	3	0.91	0.70–1.18	23.8	0.23		2	0.46	0.25–0.85	44.0	0.18	
No. of cases												
≥ 1000	2	1.19	0.85–1.66	90.0	<0.01	0.16	1	0.71	0.58–0.87	NA	NA	0.21
100–1000	4	1.00	0.82–1.21	0.0	0.57		4	0.83	0.65–1.06	24.0	0.27	
≤ 100	6	0.79	0.60–1.04	46.0	0.10		2	0.28	0.15–0.55	0.0	0.84	
Duration of follow-up years												
≥ 10 years	5	1.02	0.84–1.25	86.0	<0.01	0.63	NA	NA	NA	NA	NA	NA
< 10 years	3	0.89	0.62–1.29	0.0	0.98		NA	NA	NA	NA	NA	NA
Age												
≥ 70	4	1.00	0.96–1.03	0.0	0.80	0.45	3	0.61	0.34–1.09	68.0	0.04	0.66
< 70	5	0.89	0.66–1.02	83.0	<0.01		4	0.76	0.57–1.01	52.0	0.10	
Adjustment for confounders												
Smoking												
Yes	5	1.06	0.87–1.29	85.0	<0.01	0.21	2	0.87	0.67–1.12	0.0	0.50	0.32
No	3	0.78	0.55–1.11	0.0	0.52		5	0.60	0.42–0.88	60.0	0.04	
Alcohol												
Yes	4	1.10	0.84–1.43	81.0	<0.01	0.18	2	0.87	0.67–1.12	0.0	0.50	0.32
No	4	0.93	0.79–1.10	16.0	0.31		5	0.60	0.42–0.88	60.0	0.04	
BMI												
Yes	5	1.00	0.77–1.29	79.0	<0.01	0.71	4	0.80	0.67–0.95	10.0	0.34	0.05
No	3	0.99	0.96–1.03	0.0	0.83		3	0.42	0.25–0.71	35.0	0.21	
Physical activity												
Yes	6	1.03	0.82–1.29	75.0	<0.01	0.47	2	0.72	0.52–1.00	37.0	0.21	0.93
No	2	0.82	0.47–1.43	63.0	0.10		5	0.67	0.45–1.01	65.0	0.02	
Sunlight exposure												
Yes	0	NA	NA	NA	NA	NA	2	0.74	0.63–0.88	0.0	0.43	0.67
No	8	1.01	0.84–1.20	80.0	<0.01		5	0.62	0.38–1.01	67.0	0.02	
Total energy intake												
Yes	5	1.11	0.91–1.35	86.0	<0.01	0.05	0	NA	NA	NA	NA	NA
No	3	0.69	0.49–0.96	0.0	0.45		7	0.71	0.55–0.91	54.0	0.04	
Calcium and vitamin D supplementation												
Yes	5	1.12	0.92–1.36	86.0	<0.01	0.04	0	NA	NA	NA	NA	NA
No	3	0.69	0.50–0.95	0.0	0.47		7	0.71	0.55–0.91	54.0	0.04	

Abbreviations: *N*, the number of studies; *CI*, confidence interval; *OR*, odds ratio; *RR*, relative risk; *NA*, not applicable; *BMI*, body mass index. *P*^a^, heterogeneity within each subgroup; *P*^b^, heterogeneity between subgroups with meta-regression analysis

#### Dose-response analysis

**Fig. 4:**
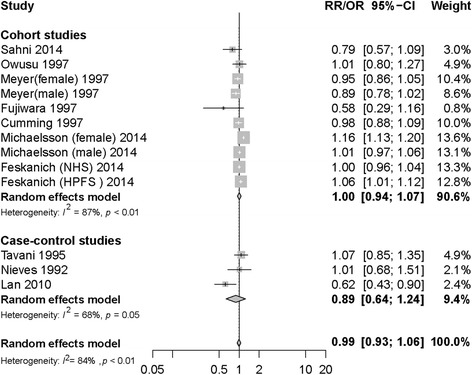
Milk consumption and risk of hip fracture. The summary relative risk per 200 g/d by using random-effects models

**Fig. 5:**
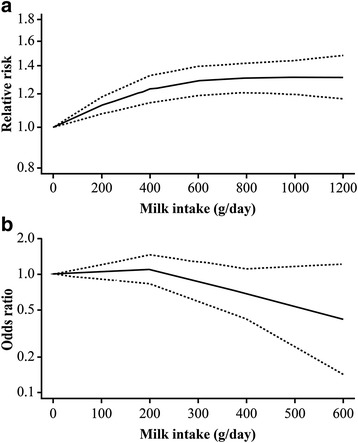
Dose-response relationship between milk consumption and risk of hip fracture

The relationship between milk consumption and hip fracture risk was further quantified via dose-response analysis for milk consumption. The summary RR for an increased milk consumption of 200 g/day was 1.00 (95% CI: 0.94–1.07), with significant heterogeneity among cohort studies (I^2^ = 87%, P_heterogeneity_ < 0.01, Fig. 4). The dose-response associations between milk consumption and risk of hip fracture in the cohort studies (*n* = 7) [12, 13, 16, 33, 34, 37, 42] are shown in Fig. 5a. There was a nonlinear positive association between milk consumption and hip fracture risk (P_nonlinerity_ < 0.01), with a rapid increase in risk when milk consumption increased from 0 to 600 g/d; there was no further increase in risk with milk consumption between 600 and 1200 g/d.

The summary OR for increasing milk consumption by 200 g/day was 0.89 (95% CI: 0.64–1.24), with significant heterogeneity among case-control studies (I^2^ = 68%, P_heterogeneity_ = 0.05, Fig. 4). Dose-response meta-analysis of the association between milk consumption and hip fracture risk in case-control studies (*n* = 3) [30, 39, 41] suggested a nonlinear association between milk consumption and hip fracture risk (P_nonlinerity_ = 0.28), with a reduction in risk with milk consumption of 200–600 g/d. However, the confidence intervals were wide for all outcomes (Fig. 5b).

### Other dairy product intake and hip fracture risk

Consumption of yogurt (*n* = 3) and cheese (*n* = 3) reduced hip fracture risk, total dairy products (*n* = 2) and cream (*n* = 1) showed no association with hip fracture risk in cohort studies for the highest versus lowest category (Fig. 2a). Consumption of total dairy products (*n* = 1), yogurt (*n* = 1), and cheese (*n* = 3) in case-control studies showed no association with hip fracture risk (Fig. 2b). No additional contour-enhanced funnel plots, subgroup, or dose-response analyses for total dairy products, yogurt, cheese, and cream could be performed because of the limited results reported in the included studies.

## Discussion

In this meta-analysis, a higher intake of yogurt and cheese was associated with a significant reduction in hip fracture risk as compared to low intake in cohort studies, and there was no overall association reported in case-control studies. Cohort and case-control studies reported no overall associations between total dairy products and cream, and hip fracture risk. Milk consumption was associated with a non-significant 9% lower hip fracture risk for highest versus lowest consumption in cohort studies. Nevertheless, the results of the case-control studies showed a significant 29% reduction in hip fracture risk for highest versus lowest consumption. The association between milk consumption and hip fracture risk remained unchanged when stratified by multiple study characteristics. Our finding for highest versus lowest milk consumption is consistent with the results from previous meta-analyses [14, 15]. Highest versus lowest analysis is limited, owing to differences in both the level, and range of milk consumption between the included studies, which may contribute to heterogeneity in the results. However, we further refined the precision of the risk estimates by applying the dose-response analysis for milk consumption, which may be important to guide recommendations for milk consumption with regard to risk reduction. Meanwhile, it is important to define and evaluate the potential threshold effects between milk consumption and hip fracture risk. In the nonlinear dose-response analysis, a low threshold of 200 g/day may have beneficial effects, whereas there is a degree of uncertainty with higher milk consumption.

Dairy products have a complicated influence on human health, and evidence on the impact of dairy products on hip fracture development remains inconsistent. Some previous studies indicated that dairy products might be beneficial for the prevention of hip fracture, as they contain calcium and vitamin D. Dairy products are often fortified with vitamin D in the United States, which is essential for the absorption of calcium and bone health [43]. A previous study has shown that vitamin D supplementation, with or without calcium, may have only minor effects on fracture risk among community-dwelling individuals [44]. Nonetheless, Chapuy et al., [45] showed that hip fracture risk was reduced with vitamin D and calcium supplementation among elderly women (mean age 84 years) who had very low vitamin D levels, with concurrent low dietary intake of calcium. Supplementation with vitamin D and calcium is a health-seeking behavior, which could be an important confounder.

Other studies argue that D-galactose in milk might promote oxidative stress and inflammation, which in turn influences the risk of fracture and mortality [13]. D-galactose is known to cause oxidative stress, aging, and inflammation. Milk contains high levels of lactose and galactose, while cheese and yogurt contain lower or non-existent levels. Previous cohort studies observed that milk consumption had a positive relationship with concentrations of marks for oxidative stress and inflammation.

These data suggest that higher milk, yogurt, and cheese consumption may contribute to a lower hip fracture risk, although the results with respect to milk consumption were not statistically significant in cohort studies. In contrast to high milk consumption, high yogurt and cheese consumption was associated with a significant 25%–32% lower risk of hip fracture, for the highest versus lowest consumption in cohort studies. Unlike milk, yogurt and cheese contain probiotics, which can improve bone formation, increase bone mass density and prevent bone loss. A study by Lei. et al., used probiotics to treat elderly patients with hip fracture on functional recovery [46].

This meta-analysis involved a larger number of cases to enhance the statistical power. Subgroup and dose-response analyses were performed to explore the heterogeneity of sources, used contour-enhanced funnel plots to display publication bias, and performed sensitivity analyses to test the robustness of the risk estimates. This meta-analysis is the first meta-analysis to evaluate the relationship between different types of dairy product consumption and hip fracture risk.

Our meta-analysis was subject to some limitations that may have affected the results. First, it is possible that the link between dairy product consumption and hip fracture risk could be interpreted within measurement errors in the dietary assessment. Food frequency questionnaires can be limited by errors in reporting and by incomplete assessment of all sources of dairy product consumption, which can lead to misclassification of exposure and weaken the association towards the null. Second, compared to cohort studies, case-control studies may have recall and selection bias. Dairy products and their possible role in bone health were widely discussed. The public debate might produce a bias in collecting dietary data among patients. Due to recall error, or dietary changes after hip fracture, participants are likely to have provided the current dietary data as a proxy for the previous diet. Cases reported a significant decrease in the frequency of dairy product consumption after the hip fracture, and it was even more obvious when the cases that provided a deliberate change in their dietary date were excluded. However, this was not evident with the control participants [47]. Indeed, inconsistent results between cohort and case-control studies were found, which might be explained by publication bias that was detected in the case-control studies. Publication bias refers to the idea that studies with positive results are more likely to be submitted for publication than those with negative results, which leads to misleading conclusions in meta-analyses. Third, differential loss to follow-up is a well-known source of bias in cohort studies, and the direction of that bias is hard to predict. Identification of fracture events may be an additional reason in cohort studies. Furthermore, the included studies may be limited by their use of differing means of assessing and measuring exposure and outcome, thereby impacting study quality scores. Ascertainment of hip fractures was partly or completely assumed by self-report in the included studies, which is also a source of bias. Mortality after hip fracture is high and a large proportion of persons who suffer a hip fracture are discharged to nursing facilities. Loss to follow-up and self-report are two important factors that increase the probability of a hip fracture not being reported, which could have affected the results. Fourth, high heterogeneity across studies was observed in this meta-analysis and baseline characteristics and adjustment for confounders also affected the results. Analyses of high versus low consumption were limited because of the different units (glasses/day, times/week, gram/day, servings/week) of dairy product consumption reported between studies, which may explain some heterogeneity in the results. Meta-regression analyses were used to explore potential sources of heterogeneity in our meta-analysis results, such as whether the studies adjusted for calcium and vitamin D supplementation, BMI, total energy intake, region, and study quality. Energy intake may increase when dairy product consumption increases, and BMI increases as total energy intake increases [48]. Several previous meta-analyses have suggested that BMI is inversely associated with hip fracture risk [49–51]. Meanwhile, a recent large, prospective, population-based study indicated that participants with BMI ≥ 25 kg/m^2^ had a reduced risk of hip fracture and patients with BMI < 22 kg/m^2^ had an increased risk compared with those with BMI between 22 and 24.9 kg/m^2^ [52], these sources of heterogeneity may have substantially influenced the results. Finally, only a small number of studies were available for the effects of total dairy products, cheese, yogurt, and cream consumption on hip fracture risk. Therefore, there was limited statistical power in the subgroup and dose-response analyses for our meta-analysis.

## Conclusions

The conclusions of this meta-analysis were discordant. Milk consumption was found to be associated with an average 29% decrease in hip fracture risk in the included case-control studies. Meanwhile, recall bias or other possible bias could be a major influence on the findings in the case-control studies. The inconsistent findings for cohort studies indicate that there is no consistent evidence on the association between milk consumption and the risk of hip fracture. Therefore, we were unable to draw any conclusion from the estimates on the association between milk consumption and hip fracture risk. Consumption of other dairy products, yogurt and cheese intake was associated with lower risk of hip fracture in cohort studies, and total dairy products and cream was not significantly associated with hip fracture risk.

## Additional files

Additional file 1: Description: Search Phrases for a) PubMed, and b) EMBASE. (DOC 37 kb)

Additional file 2: Title: Selection procedure for inclusion and exclusion of the studies. (DOC 50 kb)

## Abbreviations

BMI: Body mass index
CI: Confidence interval
FFQ: food frequency questionnaire
OR: odds ratio
RR: relative risk
the ICD-10: International Classification of Diseases, 10th revision
