# Supplementary material for: Dairy product consumption and risk of hip fracture: a systematic review and meta-analysis
Source: BMC Public Health. 2018 Jan 22;18:165. doi: 10.1186/s12889-018-5041-5 (PMC5778815; doi:10.1186/s12889-018-5041-5)
Supplement: Supplementary file 1 — Description: Search Phrases for a) PubMed, and b) EMBASE. (DOC 37 kb) [file 12889_2018_5041_MOESM1_ESM.doc]

**Additional file 1. Search Phrases for a) PubMed, and b）EMBASE**

a) Search strategy for PubMed (Publication date to 2017/4/17)

| 1. "Hip Fractures"[Mesh] |
| --- |
| 1. ((((hip fracture*[Title/Abstract]) OR Subtrochanteric Fracture*[Title/Abstract]) OR Trochanteric Fracture*[Title/Abstract]) OR Intertrochanteric Fracture*[Title/Abstract]) OR Femoral Neck Fracture*[Title/Abstract] |
| 1. 1 OR 2 |
| 1. "Dairy Products"[Mesh] |
| 1. ((((((dairy product*[Title/Abstract]) OR milk*[Title/Abstract]) OR cheese*[Title/Abstract]) OR yoghurt*[Title/Abstract]) OR butter*[Title/Abstract]) OR ice cream*[Title/Abstract]) OR dairy*[Title/Abstract] |
| 1. 4 OR 5 |
| 1. 3 AND 6 |

b) Search strategy for EMBASE (Publication date to 2017/4/17)

| 1. 'hip fracture'/exp |
| --- |
| 1. ('hip fracture' OR 'Subtrochanteric Fracture' OR 'Trochanteric Fracture' OR 'Intertrochanteric Fracture' OR 'Femoral Neck Fracture'):ab,ti |
| 1. 1 OR 2 |
| 1. 'dairy product'/exp |
| 1. dairy*:ab,ti OR 'dairy product*':ab,ti OR milk*:ab,ti OR cheese*:ab,ti OR yoghurt*:ab,ti OR butter*:ab,ti OR ice:ab,ti AND cream*:ab,ti |
| 1. 4 OR 5 |
| 1. 3 AND 6 |
