# Supplementary material for: Dairy product consumption and risk of hip fracture: a systematic review and meta-analysis
Source: BMC Public Health. 2018 Jan 22;18:165. doi: 10.1186/s12889-018-5041-5 (PMC5778815; doi:10.1186/s12889-018-5041-5)
Supplement: Supplementary file 2 — Title: Selection procedure for inclusion and exclusion of the studies. (DOC 50 kb) [file 12889_2018_5041_MOESM2_ESM.doc]

|  | **Reference list no.** |
| --- | --- |
| Studies included in the meta-analysis (n = 18) | 1-18 |
| Additional relevant studies identified through manual reference search (n = 1) | 12 |
| Studies excluded from meta-analysis, and reasons are listed below (n = 22) | 19-40 |
| **Reason for exclusion** |  |
| Publication with cases included in a selected study (n = 6) | 19-24 |
| Shared an identical population (n = 2) | 25-26 |
| Letters or comments (n = 3) | 27-29 |
| Reviews or meta-analyses (n = 2) | 30-31 |
| No sufficient data for analysis (n = 9) | 32-40 |

**Additional file 2. Selection procedure for inclusion and exclusion of the studies**

**REFERENCES LIST**

[1] Sahni S, Mangano KM, Tucker KL, Kiel DP, Casey VA, Hannan MT. Protective association of milk intake on the risk of hip fracture: results from the Framingham Original Cohort. J Bone Miner Res. 2014;29 (8):1756-62.

[2] Michaelsson K, Wolk A, Langenskiold S, Basu S, Warensjo Lemming E, Melhus H, Byberg L. Milk intake and risk of mortality and fractures in women and men: cohort studies. Bmj, 2014;349:g6015.

[3] Feskanich D, Bischoff-Ferrari HA, Frazier AL, Willett WC. Milk consumption during teenage years and risk of hip fractures in older adults. JAMA Pediatr. 2014;168 (1):54-60.

[4] Feart C, Lorrain S, Ginder Coupez V, Samieri C, Letenneur L, Paineau D, Barberger-Gateau P. Adherence to a Mediterranean diet and risk of fractures in French older persons. Osteoporos Int. 2013;24(12):3031-41.

[5] Benetou V, Orfanos P, Zylis D, Sieri S, Contiero P, Tumino R, Giurdanella MC, Peeters PH, Linseisen J, Nieters A, Boeing H, Weikert C, Pettersson U, Johansson I, Bueno-de-Mesquita HB, Dorronsoro M, Boffetta P, Trichopoulou A. Diet and hip fractures among elderly Europeans in the EPIC cohort. Eur J Clin Nutr. 2011;65(1):132-9.

[6] Lan TY, Hou SM, Chen CY, Chang WC, Lin J, Lin CC, Liu WJ, Shih TF, Tai TY. Risk factors for hip fracture in older adults: a case-control study in Taiwan. Osteoporos Int. 2010;21(5):773-84.

[7] Jha RM, Mithal A, Malhotra N, Brown EM. Pilot case-control investigation of risk factors for hip fractures in the urban Indian population. BMC Musculoskelet Disord. 2010;11:49.

[8] Jitapunkul S, Yuktananandana P, Parkpian V. Risk factors of hip fracture among Thai female patients. J Med Assoc Thai. 2001;84 (11):1576-81.

[9] Kanis J, Johnell O, Gullberg B, Allander E, Elffors L, Ranstam J, Dequeker J, Dilsen G, Gennari C, Vaz AL, Lyritis G, Mazzuoli G, Miravet L, Passeri M, Perez Cano R, Rapado A, Ribot C. Risk factors for hip fracture in men from southern Europe: the MEDOS study. Mediterranean Osteoporosis Study. Osteoporos Int. 1999;9(1):45-54.

[10] Owusu W, Willett WC, Feskanich D, Ascherio A, Spiegelman D, Colditz GA. Calcium intake and the incidence of forearm and hip fractures among men. J Nutr. 1997;127(9):1782-87.

[11] Fujiwara S, Kasagi F, Yamada M, Kodama K. Risk factors for hip fracture in a Japanese cohort. J Bone Miner Res. 1997;12(7):998-1004.

[12] Cumming RG, Cummings SR, Nevitt MC, Scott J, Ensrud KE, Vogt TM, Fox K. Calcium intake and fracture risk: results from the study of osteoporotic fractures. Am J Epidemiol. 1997;145(10):926-34.

[13] Johnell O, Gullberg B, Kanis JA, Allander E, Elffors L, Dequeker J, Dilsen G, Gennari C, Vaz AL, Lyritis G, Mazzuoli G, Miravet L, Passeri M, Cano RP, Rapado A, Ribot C. Risk factors for hip fracture in european women: The MEDOS study. Journal of Bone and Mineral Research. 1995;10(11):1802-15.

[14] Meyer HE, Pedersen JI, Løken EB, Tverdal A. Dietary factors and the incidence of hip fracture in middle-aged Norwegians: A prospective study. American Journal of Epidemiology. 1997;145(2):117-23.

[15] Tavani A, Negri E, La Vecchia C. Calcium, dairy products, and the risk of hip fracture in women in northern Italy. Epidemiology. 1995;6(5):554-7.

[16] Cumming RG, Klineberg RJ. Case-control study of risk factors for hip fractures in the elderly. Am J Epidemiol. 1994;139(5):493-503.

[17] Nieves JW, Grisso JA, Kelsey JL. A case-control study of hip fracture: evaluation of selected dietary variables and teenage physical activity. Osteoporos Int. 1992;2(3):122-7.

[18] Sahni S, Tucker KL, Kiel DP, Quach L, Casey V, Hannan MT. Milk intake is associated with lower risk of hip fracture in older men & women: The framingham original cohort. FASEB Journal. 2013;27.

[19] Kanis JA, Johansson H, Oden A, De Laet C, Johnell O, Eisman JA, Mc Closkey E, Mellstrom D, Pols H, Reeve J, Silman A, Tenenhouse A. A meta-analysis of milk intake and fracture risk: low utility for case finding. Osteoporos Int. 2005;16(7):799-804.

[20] Feskanich D, Willett WC, Colditz GA. Calcium, vitamin D, milk consumption, and hip fractures: a prospective study among postmenopausal women. Am J Clin Nutr. 2003;77(2):504-11.

[21] Feskanich D, Willett WC, Stampfer MJ, Colditz GA. Milk, dietary calcium, and bone fractures in women: a 12-year prospective study. Am J Public Health. 1997;87(6):992-7.

[22] Unay K, Demircay E, Akan K, Sener N. [Risk factors for osteoporosis in women having hip fractures after 60 years of age]. Acta Orthop Traumatol Turc. 2005;39(4):295-9.

[23] Turner LW, Wang MQ, Fu Q. Risk factors for hip fracture among southern older women. South Med J. 1998;91(6):533-40.

[24] Turner LW, Fu Q, Taylor JE, Wang MQ. Osteoporotic fracture among older U.S. women: risk factors quantified. J Aging Health. 1998;10(3):372-91.

[25] Feskanich D, Willett WC, Stampfer MJ, Colditz GA: Milk, dietary calcium, and bone fractures in women: a 12-year prospective study. American journal of public health 1997, 87(6):992-997.

[26] Sahni S, Tucker KL, Kiel DP, Quach L, Casey VA, Hannan MT. Erratum: Milk and yogurt consumption are linked with higher bone mineral density but not with hip fracture: The Framingham offspring study. Archives of Osteoporosis. 2013;8(1-2) DOI: 10.1007/s11657-013-0119-2.

[27] Heaney RP. Early-life milk and late-life fracture. JAMA Pediatr. 2014;168(7):682-3.

[28] Howland RH. Early-life milk and late-life fracture. JAMA Pediatr. 2014;168(7):683.

[29] Feskanich D, Willett WC. Early-life milk and late-life fracture: Reply. JAMA Pediatrics. 2014;168(7):683-4.

[30] Bischoff-Ferrari HA, Dawson-Hughes B, Baron JA, Kanis JA, Orav EJ, Staehelin HB, Kiel DP, Burckhardt P, Henschkowski J, Spiegelman D, Li R, Wong JB, Feskanich D, Willett WC. Milk intake and risk of hip fracture in men and women: a meta-analysis of prospective cohort studies. J Bone Miner Res. 2011;26(4):833-9.

[31] Cumming RG, Nevitt MC. Calcium for prevention of osteoporotic fractures in postmenopausal women. J Bone Miner Res. 1997;12(9):1321-9.

[32] Pripp AH, Dahl OE. The population attributable risk of nutrition and lifestyle on hip fractures. Hip Int. 2015;25(3):277-81.

[33] Wlodarek D, Glabska D, Kolota A, Adamczyk P, Czekajlo A, Grzeszczak W, Drozdzowska B, Pluskiewicz W. Calcium intake and osteoporosis: the influence of calcium intake from dairy products on hip bone mineral density and fracture incidence - a population-based study in women over 55 years of age. Public Health Nutr. 2014;17(2):383-9.

[34] Zeng FF, Fan F, Xue WQ, Xie HL, Wu BH, Tu SL, Ouyang WF, Chen YM. The association of red meat, poultry, and egg consumption with risk of hip fractures in elderly Chinese: A case-control study. Bone. 2013;56(2):242-8.

[35] Rizzoli R. Dairy products: A way towards better bone health. Osteoporosis International. 2013;24(1):S390.

[36] Feart C, Letenneur L, Lorrain S, Ginder V, Samieri C, Paineau D, Barberger-Gateau P. Association between the mediterranean diet adherence and the risk of hip fracture in elderly people. Osteoporosis International. 2012;23:S200-1.

[37] Sahni S, Hannan MT, Quach L, Casey VA, Tucker KL. Is dairy intake associated with the risk of hip fracture?: 12-y follow-up from the Framingham Offspring Study. FASEB Journal. 2011;25.

[38] Rizzoli R. Dairy products and bone: Devil or angels?. Osteoporosis International. 2010;21:S399.

[39] Grønskag AB, Forsmo S, Romundstad P, Langhammer A, Schei B. Dairy products and hip fracture risk among elderly women in Norway - The hunt study. Osteoporosis International. 2010;21:S94-5.

[40] Turner LW, Hunt S, Kendrick O, Eddy J. Dairy-product intake and hip fracture among older women: issues for health behavior. Psychol Rep. 1999;85(2):423-30.
